# Identifying Cancer Specific Functionally Relevant miRNAs from Gene Expression and miRNA-to-Gene Networks Using Regularized Regression

**DOI:** 10.1371/journal.pone.0073168

**Published:** 2013-10-02

**Authors:** Aziz M. Mezlini, Bo Wang, Amit Deshwar, Quaid Morris, Anna Goldenberg

**Affiliations:** 1 Department of Computer Science, University of Toronto, Toronto, Ontario, Canada; 2 Genetics and Genome Biology, SickKids Research Institute, Toronto, Ontario, Canada; 3 Edward S. Rogers Sr. Department of Electrical and Computer Engineering, University of Toronto, Toronto, Ontario, Canada; 4 Donnelly Centre, University of Toronto, Toronto, Ontario, Canada; The University of Hong Kong, China

## Abstract

Identifying microRNA signatures for the different types and subtypes of cancer can result in improved detection, characterization and understanding of cancer and move us towards more personalized treatment strategies. However, using microRNA's differential expression (tumour versus normal) to determine these signatures may lead to inaccurate predictions and low interpretability because of the noisy nature of miRNA expression data. We present a method for the selection of biologically active microRNAs using gene expression data and microRNA-to-gene interaction network. Our method is based on a linear regression with an elastic net regularization. Our simulations show that, with our method, the active miRNAs can be detected with high accuracy and our approach is robust to high levels of noise and missing information. Furthermore, our results on real datasets for glioblastoma and prostate cancer are confirmed by microRNA expression measurements. Our method leads to the selection of potentially functionally important microRNAs. The associations of some of our identified miRNAs with cancer mechanisms are already confirmed in other studies (hypoxia related hsa-mir-210 and apoptosis-related hsa-mir-296-5p). We have also identified additional miRNAs that were not previously studied in the context of cancer but are coherently predicted as active by our method and may warrant further investigation. The code is available in Matlab and R and can be downloaded on http://www.cs.toronto.edu/goldenberg/Anna_Goldenberg/Current_Research.html.

## Introduction

As a highly conserved major factor of post-transcriptional regulation, microRNAs are believed to have a significant impact on gene expression and therefore on most biological mechanisms and functions. The link between miRNAs and cancer has been the subject of several recent studies and reviews [1]–[4]. The belief that miRNA's profile change is not only a bystander consequence of cancer but may also have an active role in it, is consolidated by several observations. First, miRNA expression data shows coherent and drastic changes in levels of expression between tumor tissues and their healthy counterparts. Second, it has been observed that many miRNAs are located on the genome in regions that are prone to CNVs/deletions in cancer [5]. Finally and most importantly, many miRNAs have been shown to have a direct effect on cancer-related mechanisms such as apoptosis [6], proliferation [7], angiogenesis [8] and metastasis [9].

Differential analysis of miRNA expression profiles in cancer vs healthy tissue alone may lead to a large number of false positives due to the noisy nature of miRNA expression data. Additionally, we have poor knowledge of how much deviation in expression of a particular miRNA can induce functionally relevant changes in gene expression. It is possible that the gene expression profile is highly sensitive to even small changes in some miRNAs' quantities while drastic changes in other miRNAs do not have significant impact on it.

Previous methods for detecting important miRNAs in cancer make a series of key assumptions. For example, RegulatorInference [10] is a very recent method that aims to find the active miRNAs via a regression approach. It implicitly assumes that all genes are affected by copy number variations in the same linear fashion and that the effect of a miRNA on a target gene is linear in the number of binding sites. Additionally, RegulatorInference uses miRNA expression data to preselect potentially active miRNAs, though this step is optional. In [11], gene expression, miRNA expression and the gene-gene interaction network are used to construct a putative complex miRNA-target influence network with miRNA influence coefficients that are computed based on miRNA direct/indirect effect on genes and gene-gene annotated interactions.

In this paper, we present a method for predicting functionally relevant miRNAs from differential gene expression data using miRNA-gene interaction information and mRNA expression only. Contrary to previous methods, we do not use miRNA expression data in our predictions and we make no assumptions on how different miRNAs, CNVs or interactions may influence different genes. The idea is to select a set of miRNAs responsible for most of the changes observed in gene expression based on prior knowledge of existent miRNA-gene interactions with no additional assumptions on the strengths of these interactions. Our approach is based on a regression model for the gene differential expression. We added an elastic net regularization [12] to avoid overfitting the high level of noise in the data and select a minimal set of active miRNAs. The miRNAs differential expression data is only used for validation purposes. To the extent of our knowledge, this is the first approach to determine active miRNAs in cancer from gene expression data only (no miRNA data) and therefore we can assess our performance using differential miRNA expression data from the same patients.

Our simulations show that our method is robust to several types of noise and missing data and that it is able to predict the relevant miRNAs as long as the input gene expression changes are, at least, partially due to miRNAs. We used our data on 157 glioblastoma patients and 111 prostate cancer patients and predicted several relevant miRNAs effects which were confirmed by miRNA expression measurements on these same patients.

Our results on real data identified miRNAs whose roles were previously validated in cancer such as mir-210 and mir-296-5p, along with other potentially important miRNAs: mir-1, mir-154, mir-339-3p, mir-539, mir-561, mir-607 in glioblastoma and mir-143*, mir-30c-2*, mir-330-3p, mir-526b and mir-939 in prostate cancer.

## Methods

### 1 Data pre-processing

A large proportion of the variability in gene/miRNA expression measurements across different tumour samples is due to the different levels of contamination with healthy cells. Typically 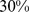 to 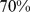 of a tumour tissue consists of healthy cells whose gene/miRNA expression signal will perturb the cancer signal to a different extent for the different samples (patients).

We use ISOpure [13] to extract the purified cancer signal for all the patients. This gives a better coherence between cancer patients and more accurate results for miRNA prediction.

### 2 Normalized differential expression computation

After we purify the tumour data, we take the log of the gene measurements in patients and in controls. Then, for every patient we compute the normalized differential expression for one gene or one miRNA according to [14]:
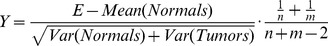
(1)Where E and Y are respectively the original and the normalized gene expression for the patient, n and m are respectively the number of healthy controls and cancer patients, 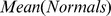 and 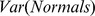 are the average and the variance of expression for that gene in the healthy controls. The term 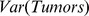 penalizes the genes having high variance between cancer patients. The same normalization is used for the miRNA data to obtain differential miRNA expression for tumour vs healthy samples when we use miRNA expression for validation purposes.

### 3 Selecting active miRNAs

**Figure 1:**
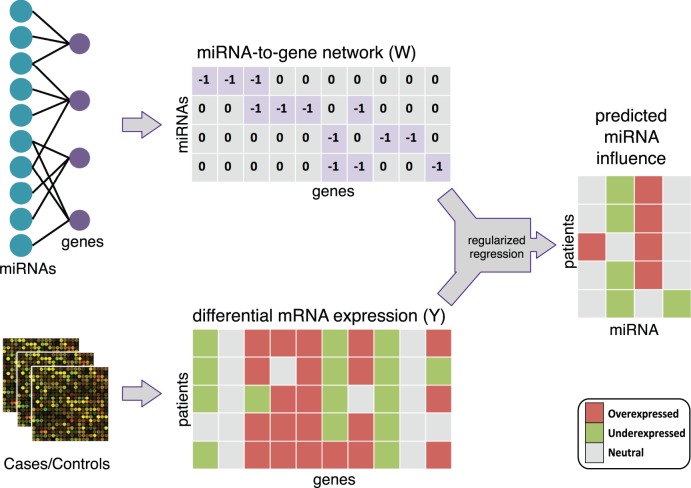
Flow chart of the active miRNAs prediction approach. We can predict the active miRNAs for each individual patient, then we investigate the coherence of the active miRNAs across patients.

We determine the list of active miRNAs by selecting miRNAs that would have had to be active to observe the resulting mRNA expression change pattern across the genome. More specifically, we model the genome-wide change in mRNA expression levels 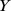 as a sum of unknown miRNA influences 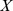. The influences have non-zero contribution for all miRNAs that target a given gene. We use the miRNA-to-gene network for gene target information and represent it as a matrix 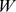. We obtained the network from the European Bioinformatics Institute (ww.ebi.ac.uk/enright-srv/microcosm/cgi-bin/targets/v5/download. pl) that provided a set of target genes for every miRNA (711 miRNAs, 16799 genes and 470,000 edges). If there is no discernible effect of the miRNA expression change on the targets' expression change, the influence is estimated to be zero. The miRNA influences 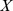 are estimated on a per patient basis. If the predicted miRNA influence is non-zero and has the same sign across majority of the patients, we presume that the given miRNA is active and may be an important factor for a given disease. The flow chart for our method is captured in Figure 1 and the formulation is given below:
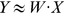
(2)


Matrix 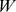 of microRNA-gene interactions is defined as follows:
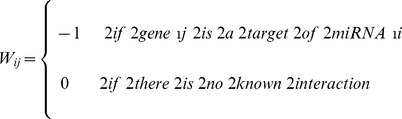
(3)


The negative values in the 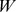 matrix indicate that the miRNAs usually down-regulate gene expression. Since the information on how much a particular miRNA affects a given gene is not available, the coefficients in the solution 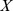 represent the “influence” of miRNAs on the gene expression profile rather than miRNAs differential expression (i.e. how much of the change in gene expression of miRNA targets can be attributed to that particular miRNA). 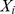 indicates two things: 1) whether miRNA 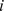 was active (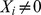); and 2) in what way it influenced the expression, i.e. whether the target genes are over-expressed in cancerous tissue compared to normal tissue indicating that miRNA levels were depleted (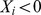) or under-expressed indicating the over-expression of the given miRNA (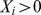).

If the influence 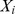 of an active miRNA is of the same sign with the given patient's observed miRNA differential expression, we say that the 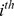 miRNA prediction is validated by the miRNAs measurements.

Since we want to avoid overfitting and encourage models with a small number of active miRNAs (simpler models with only a few active miRNAs are easier to interpret and are biologically more relevant), we use an elastic net type penalty. The resulting objective function to minimize is:
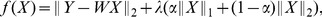
(4)where 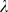 and 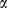 are the elastic net parameters, the choice of parameters is discussed below. Elastic net penalty is chosen here because it is able to avoid some of the limitations we may encounter using a simple 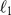 penalty as in [10]. For example, when we have highly correlated variables (miRNAs with a lot of targets in common) an 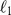 penalty will tend to select one variable at random and ignore the others, whereas elastic net will select all the relevant variables.

### 4 Choice of parameters (cross validation)

The parameters of the elastic net define the sparsity of the solution: the number of miRNAs that are predicted to be actively driving the genes differential expression. To determine 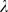, we used the cross validation framework proposed in [12] (Implementation is available in R glmnet package). Acceptable results are obtained with 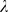 corresponding to the minimal predictive error using 10-fold cross validation. Choosing the highest 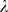 corresponding to a prediction error of 1 standard error plus the minimal predictive error can lead to more accurate results in simulations with lower levels of noise. However, it often gives overly sparse solutions when the prediction error has high variance and therefore we use the minimal prediction error convention in those cases. In our results, we fix the same 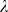 for all patients to be the average selected value across patients. For the choice of 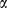 we repeated the whole experiment (simulation and real data) with different values (0.1, 0.25, 0.5) and no major difference was observed for the final predicted miRNAs, so we set our 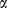 to 0.25 for all experiments.

## Results

### 1 Simulations and robustness to noise

First, we tested the ability of our method to select the correct subset of gene-expression-driving miRNAs among the set of all miRNAs, and the ability to determine the correct influences for the selected miRNAs (sign of 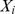 in (2)). To do that, we randomly selected 30 miRNAs to be the active miRNAs driving the gene expression changes and we assigned large positive or negative mean expression values to them (absolute value uniformly selected from 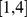 and sign obtained by unbiased coin tossing). We assigned zero mean values to all other miRNAs that are not simulated as active. We then simulated 100 patients with miRNA differential expression profiles centred around those mean values (Gaussian distribution with given mean and standard deviation 0.5). As a result, we obtain for every patient a set of miRNA differential expression measurements in which the 30 miRNAs simulated as active will be consistently differentially expressed and all other miRNAs will represents a background noise. Finally, for every patient we multiply the miRNA simulated differential expression measures by the miRNA-gene interaction network (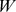 matrix in Equation 2) to obtain simulated gene differential expression measures (This step will be later referred to as the gene differential expression simulation step). The network used here is the same as the one mentioned in Section 2.3 and produced by the European Bioinformatics Institute capturing interactions between 711 miRNAs and 16799 genes, the median miRNA having around 650 potential target genes.

The goal of this experiment is to test if the problem can be solved despite the fact that the miRNAs often have hundreds of overlapping potential targets and that multiple miRNAs may have opposite effects on gene expression levels.

We ran one thousand simulations generating data as described above. We found that in 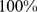 of the cases, we were able to identify the exact randomly selected subset of miRNAs driving the changes in the gene expression and correctly predict all influence signs. In practice, this means that our method works correctly for any sample in real data where miRNAs' differential expression is responsible for a relatively high proportion of the differential expression of mRNAs. This also means that the consistency in which groups of genes are over/under expressed is a strong enough signal for detecting active miRNAs even though different miRNAs may have opposite effects on common target genes.

Next we tested the robustness of our methods to the different types of noise. We examined the effect of four types of noise that could make this problem harder to solve:

- We accounted for structural noise (false negative interactions) in the miRNA-gene network by adding random edges prior to gene differential expression simulation and then using the original miRNA-gene network for the predictions, resulting some of the miRNA influence information used to generate the data was missing at the prediction stage.
- We accounted for incorrect edges (false positive interactions) in the miRNA-gene network by removing random edges prior to gene differential expression simulation. The original miRNA-gene network is still used in predictions.
- We simulated the effect of unobserved gene-gene interactions on the genes expression (we use the protein-protein interaction network).
- We increase the levels of noise in the expression data (by adding Gaussian noise with 0-mean and variance proportional to the expression levels) to account for other mechanisms altering gene-expression in cancer such as copy number variations.

**Figure 2:**
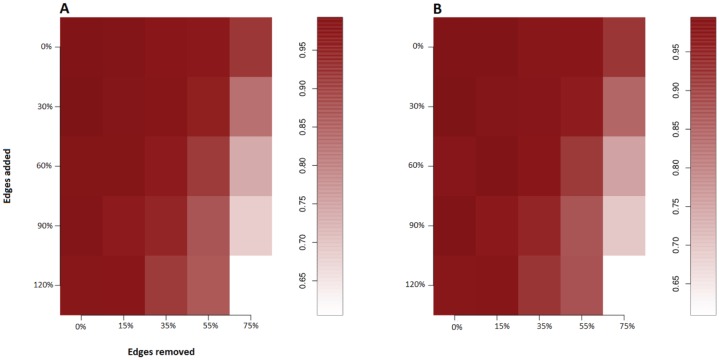
Robustness to network noise. (A) Sensitivity and (B) precision of the method in predicting the gene-expression-driving miRNAs when we varying the proportions of incorrect edges and missing edges in the miRNA-Gene interactions network (delete edges/insert new edges). All miRNA were also predicted to have the correct direction of influence (suppressor vs oncogene effect).

**Figure 3:**
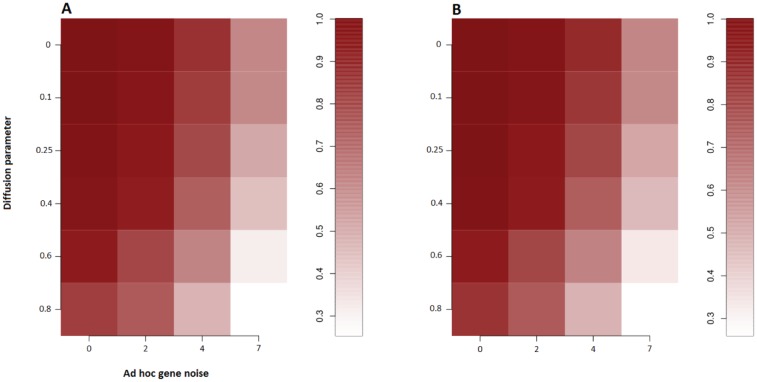
Robustness to expression noise. (A) Sensitivity and (B) precision of the method in predicting the gene-expression-driving miRNAs when varying the level of gene-interactions noise (via the diffusion parameter 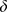) and the intensity of the noise in the differential gene expression level (via the variable 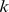). All predicted miRNAs have the correct influence signs.

Figure 2 shows the effect of modelling missing/incorrect information in the miRNA-gene network. We notice that the method is able to approximately detect the correct subset of miRNAs even when we suppose 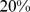 of the miRNA-gene annotated interactions are wrong (we remove 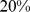 of the edges in the network graph during the gene expression simulation step) or when we suppose the annotations contain only half of the real miRNA-gene interactions (we add edges during the gene expression simulation step). There is evidence that the method is more robust to missing information (False negative edges) than to incorrect information (False positive edges) which means it is preferable to use conservative networks in this context. Figure 3, shows the robustness of the method to high levels of noise in gene expression. The gene expression noise is parametrized by the variable 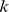 controlling the strength of the noise: for every gene, the variance of the last type of noise is 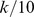 multiplied by the average level of expression (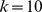 gives a Poisson type variance). Finally, the gene-gene interaction noise is controlled by the diffusion variable 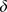 as follows:
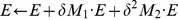
(5)


Where 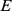 is the gene differential expression vector, 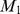 is the gene-gene direct interaction matrix and 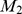 is the matrix with second order gene-gene interactions (second order neighbours in the gene-gene interactions network representation). This means a gene expression affects to a certain degree the expression of interacting genes (direct neighbours) and to a smaller degree the genes interacting with the direct neighbours (second order neighbours). The gene-gene interaction network we used here is a subset of the combined human network downloaded from Biogrid website. It captures information for the 16,799 genes that are present in our miRNA-gene network and contains 85,590 edges. Figure 3 shows that the ability of our method to recover the true miRNAs is high even in the presence of other influences, such as affects of interacting genes, which our model does not account for. Our simulation results indicate that our method is robust to different kinds of noise.

### 2 Analysis of cancer data

#### 2.1 Evaluation criteria

Using our method, we predicted the functionally relevant miRNAs and their influence on gene expression from the differential gene expression data for glioblastoma multiforme and prostate cancers. Since our predictions are on a per-patient basis, we select those miRNAs that were predicted active (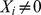) with the same direction of influence in more than 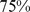 of the patients. These miRNAs are more likely to reflect a real biological mechanism common to the cancer since they were consistently predicted as active by our method in a large number of independent patients. Then we look at miRNAs' expression measurements for the same patients. If the active miRNAs' differential expressions have the same signs as their predicted influences in more than 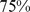 of the patients, we consider them validated by miRNA measurements.

In the miRNA differential expression data, some miRNAs are coherently over- or under-expressed across patients and some are not. We estimate the significance of our active miRNA set by computing the p-value: the probability of having as good or better validation results than our method if the miRNAs predictions were random.

We compute that p-value by first selecting a random subset of miRNAs from the data, with the set size equal to the number of miRNAs predicted as active by our method. Then, we randomly assign the direction of influence (signs) to these selected miRNAs. Finally, we estimate the proportion of these random predictions that are validated by miRNA measurements using the miRNA data corresponding to the same studied patients (a miRNA is validated if it is coherently over-expressed/under-expressed when its impact sign is respectively positive/negative). We repeat this experiment 10,000 times. The p-value is the proportion of random experiments with higher validation rate than our method's prediction. A small enough p-value indicates that the miRNAs selected by our method were not random and that there likely is a functional importance in relation to the cancer indicated by the coherence in miRNA/gene expression across patients.

#### 2.2 Glioblastoma

The glioblastoma gene expression data was produced by Broad Institute using the Affymetrix HT_HG-U133A experimental design. It contains 157 tumor cases and 10 controls. This data along with the miRNA expression we use for validation are available on the TCGA website.

**Figure 4:**
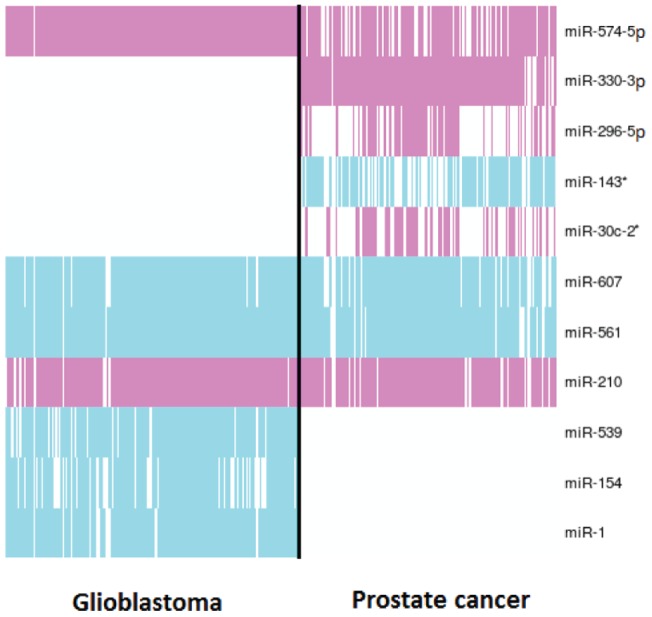
Predicted miRNAs in prostate cancer and glioblastoma patients. Pink is for over-expressed miRNAs and blue is for under-expressed. Each column represents a patient and each row a miRNA.

After purifying the gene expression data (See Section 2.1) and transforming it to normalized differential expression (See Section 2.2), we predicted 11 miRNAs active across more than 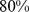 of the patients. Out of these 11 miRNAs predictions, 7 were validated by the available miRNA expression (verified in more than 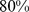 of the cases) and 2 others were verified in more than 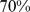 of the cases. These results correspond to a p-value of 0.0005 (see Section 3.2.1 for more details). Figure 4 shows the miRNAs coherently predicted across patients. Among the miRNAs that were predicted and confirmed, we found mir-210, mir-339-3p, mir-561, mir-1, mir-154, mir-539, mir-607.

miR-210 is inducible by hypoxia and was previously identified as an independent marker for several cancers (breast [15], pancreas [16], head and neck [17]). It is also cited as a regulator for Normoxic gene expression which is involved in tumour initiation [18]. We predicted it as active and confirmed its over-expression activity using observed data in glioblastoma.

We also predicted and confirmed the over-expression of mir-339-3p, and the under-expression of mir-1, mir-154, mir-539, mir-561 and mir-607. At present there is no knowledge about the functional roles of these miRNAs in cancer. Further validation experiments are required for a better understanding of the function of these miRNA in glioblastoma. mir-548b-5p, mir-548c-3p, mir-548c-5p were also predicted in almost all patients even though their target gene sets were very different, but they were only validated in respectively 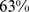, 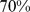, 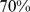 of the cases.

#### 2.3 Prostate cancer

The prostate cancer data was produced by Memorial Sloan-Kettering Cancer Center (MSKCC) and is available from GEO under the accession number GSE21032. The data contains information about 111 patients and 28 controls for which both gene and miRNA expression data are available.

After preprocessing the gene expression data to obtain the purified normalized differential expression and using our method to predict sets of active miRNAs for every patient, we selected 10 miRNAs: 7 of these miRNAs were validated using the observed miRNA expression data in more than 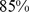 patients, confirming the direction of miRNA influence. Another miRNA: mir-574-5p was validated in 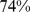 of the patients. These results correspond to a statistically significant p-value of 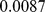.

Among the miRNAs that were predicted and confirmed, mir-210 was over-expressed similarly to our findings in the glioblastoma data, and mir-296-5p (over-expressed) was previously associated with apoptosis of androgen-independent prostate cancer cells [19] mir-143* was predicted and confirmed by the observed data as under-expressed in all patients but was not previously mentioned as a cancer marker in the literature. Similarly mir-30c-2*, mir-526b, mir-330-3p and mir-939 (all predicted and confirmed as over-expressed) were sporadically mentioned as differentially expressed miRNAs in cancer literature but with no solid experimental evidence to characterize their exact roles in cancers yet.

Finally, mir-569 and mir-607 were predicted in the majority of both prostate cancer and glioblastoma patients but were only validated in glioblastoma because they were not measured in prostate cancer data. Similarly, mir-574-5p was predicted in both datasets but its measurements were unavailable for the glioblastoma patients, therefore it was only confirmed in prostate cancer.

The overall summary of our results with highest validation rates in prostate cancer and glioblastoma are described in Figure 4.

## Discussion

In this paper we presented the first method that predicts active miRNAs in cancer from the miRNA-gene regulatory network and mRNA expression data without relying on the miRNA expression itself. We use elastic-net-regularized regression framework to make those predictions robustly. Since mRNA expression data is often readily available, our method can be used to guide the analysis of miRNAs by prioritizing them for future experiments. We validated our method using both simulation and real data coupled with miRNA expression measurements to confirm our findings. Our simulations have indicated that our method robustly identifies active miRNAs and the direction of their influence (suppressors or oncogenes) on the global gene expression patterns. Our results on real data indicated potentially important miRNAs in glioblastoma and prostate cancer, some of which were already validated in previous studies (The hypoxia related mir-210 and the prostate cancer cell apoptosis related mir-296-5p) and others for which the exact role in cancer remains to be determined.

Future improvements of our method are closely linked to better modelling of the noise in the data. For example, taking into account the expression changes that are induced by karyotypic variations or copy number variations in cancer would help isolate the effect of functionally relevant miRNAs, especially if the impact of these variations on gene expression is accurately understood and modelled in the future. Better results can also be achieved if we have a solid model of the gene-gene interactions that can alter expression independently of the miRNAs.
